# A systematic assessment of safety, tolerability and blinding effectiveness of kilohertz transcranial magnetic perturbation (kTMP)

**DOI:** 10.1088/1741-2552/ae93fc

**Published:** 2026-08-12

**Authors:** Christina M Merrick, Guy Avraham, Philipp Reber, Katheryn Thayer-Pham, Feiyang Dai, Saumya Singh, Cidnee Luu, Ian O’Kidhain, Haajar Ahmad-Ali, Angel V Peterchev, Karunesh Ganguly, Daniel Sheltraw, Richard B Ivry, Ludovica Labruna

**Affiliations:** 1 Magnetic Tides Inc, 2397 Shattuck Avenue, Suite 204, Berkeley, CA 94704, United States of America; 2 Department of Psychology, University of California Berkeley, 2121 Berkeley Way, Berkeley, CA 94720, United States of America; 3 UC Berkeley–UCSF Graduate Program in Bioengineering, Berkeley, CA 94720, United States of America; 4 Departments of Psychiatry & Behavioral Sciences, Biomedical Engineering, Electrical & Computer Engineering, and Neurosurgery, Duke University, Durham, NC 27710, United States of America; 5 Department of Neurology, University of California San Francisco, 1651 4th Street, San Francisco, CA 94158, United States of America; 6 Neurology Service, San Francisco Veterans Affairs Medical Center, San Francisco, CA 94121, United States of America

**Keywords:** kilohertz transcranial magnetic perturbation, kTMP, tolerability, non-invasive brain stimulation, neuromodulation, transcranial magnetic stimulation, safety

## Abstract

*Objective.* Non-invasive brain stimulation (NIBS) techniques are increasingly used to modulate brain activity in basic and translational research. Kilohertz transcranial magnetic perturbation (kTMP) is a recently developed NIBS approach that uses magnetic induction to generate subthreshold electric fields in the brain. kTMP has been shown to modulate cortical excitability while producing no perceptible sensation at the stimulation site. However, its safety and tolerability have not yet been systematically evaluated—a gap this study aims to address. *Approach.* We conducted sham-controlled experiments, within-subject comparisons, and patient feasibility studies with kTMP, entailing 433 sessions across 143 individuals. Participants rated annoyance, muscle activation, and pain on a 0–10 scale after each session. With primary motor cortex (M1) as the target, we compared active stimulation (∼8 V m^−1^ cortical field) to sham (0 V m^−1^) in healthy adults and chronic stroke patients. In healthy adults, we compared active vs. sham stimulation applied to dorsolateral prefrontal cortex, superior temporal gyrus, and cerebellum. Additional datasets examined tolerability across active kTMP parameters and multi-session feasibility in stroke patients. Safety monitoring included continuous observation for abnormal motor activity and EMG recording in initial experiments. *Main results.* No device-related adverse reactions occurred across 433 sessions. EMG monitoring revealed no artifacts, and no participants exhibited involuntary muscle contractions or signs of abnormal cortical excitation. kTMP was well tolerated, with mean ratings for active and sham stimulation remaining below 1.5 (where ‘2’ indicates just-noticeable sensation). Permutation tests showed no significant active–sham differences and bootstrapped 95% confidence intervals consistently fell within the ±1 equivalence margin. When auditory masking was used, participants could not distinguish active from sham stimulation. *Significance.* kTMP achieves cortical E-fields an order of magnitude higher than conventional subthreshold tES while maintaining robust safety margins and tolerability indistinguishable from sham, supporting its use for rigorous double-blind studies and translational settings.

Trial Registration: ClinicalTrials.gov Identifier: NCT06317194.

## 1. Introduction

Over the past two decades, extensive research has advanced non-invasive brain stimulation (NIBS) as a potential intervention for neurological and psychiatric disorders [1]. Electromagnetic NIBS methods modulate neuronal excitability through externally applied electric fields (E-fields). These methods fall into two broad categories: electrical contact and magnetic induction. Electrical contact approaches, such as transcranial electrical stimulation (tES), establish cortical E-fields through scalp electrodes driven by either constant (tDCS) or time-varying (tACS) currents. Magnetic induction approaches, such as transcranial magnetic stimulation (TMS), generate cortical E-fields using current-carrying coils that induce fields via rapidly changing magnetic flux. tES and TMS techniques have been used to perturb neural activity in different brain areas, including primary motor cortex (M1), dorsolateral prefrontal cortex (DLPFC), and the cerebellum. Of great interest is the potential for these methods to promote plasticity that can be employed to improve symptoms associated with disorders such as post-stroke hemiparesis, depression, and cerebellar ataxia [2–4].

Despite this progress, existing methods face a tradeoff between efficacy and tolerability. tES produces very weak cortical E-fields—typically ∼0.2–0.5 V m^−1^ [5, 6]—because much of the applied current is shunted by the scalp and skull. Importantly, even at these low cortical E-field levels, tES can still elicit uncomfortable scalp sensations (e.g. itching, burning, pinching [7]). TMS can generate much stronger cortical E-fields, often above neuronal activation threshold, but tolerability is limited by incidental activation of peripheral nerves and muscles, (i.e. facial activation during DLPFC stimulation [8] or neck activation during cerebellar stimulation [9, 10]) as well as increased incidence of headache and discomfort at the stimulation site [11, 12]. Although both tES and TMS are generally safe [13], tolerability remains a frequent reason for treatment discontinuation [14], a challenge that is particularly problematic for multi-session protocols [15].

An ideal NIBS method would combine the high E-field amplitudes necessary for robust neuromodulation with the tolerability profile required for sustained clinical use. Kilohertz transcranial magnetic perturbation (kTMP) [16] is a novel NIBS method that addresses this challenge. Designated by the University of California, Berkeley Institutional Review Board as a non-significant risk device for use in healthy populations and individuals with chronic stroke, kTMP employs a high-current amplifier to drive a standard TMS coil positioned to deliver subthreshold E-fields at the targeted brain region. Due to excessive power demands at lower frequencies, the carrier frequency is limited to 1 kHz and above. By leveraging magnetic induction, kTMP circumvents the high impedance of the skull that limits tES, enabling cortical E-field amplitudes up to 8 V m^−1^—approximately 16–40 times higher than conventional tES—while maintaining relatively low E-fields at the scalp [17]. In contrast to the brief, discrete pulses of conventional TMS, kTMP provides a continuous waveform that can be sustained for tens of minutes. The waveform can be delivered as a pure kilohertz carrier or with amplitude modulation, allowing the high-frequency carrier to embed physiologically relevant rhythms such as low-frequency oscillations (e.g. 3 Hz) or beta activity (e.g. 20 Hz).

Initial experiments with healthy adults demonstrated that 10 min of kTMP over M1 increased cortical excitability, as measured by motor evoked potential amplitudes in a target hand muscle, relative to sham stimulation [18]. Critically, participant ratings of annoyance, pain, and incidental muscle activation were indistinguishable from sham (0 V m^-1^). The only percept consistently noted in the subjective reports was the sound produced by the system at twice the carrier frequency. This sound can be easily masked, making kTMP well suited for double-blind designs.

In these initial experiments, tolerability was assessed with a relatively modest, target E-field amplitude (2 V m^−1^), with testing limited to 10 min at a single stimulation site (M1). We have subsequently developed kTMP systems capable of producing higher E-field amplitudes and have conducted a series of experiments to evaluate both efficacy and tolerability across a wider range of stimulation parameters. To date, we have completed 607 sessions involving over 200 individuals with no device-related adverse reactions using the next generation kTMP systems. In this report we focus on the data from a subset of these sessions that involved sham-controlled experiments, direct within-subject comparisons or feasibility studies involving patients with neurological disorders. This dataset includes 298 sessions in healthy adults and 135 sessions in patients with chronic stroke. With M1 as the target, we compared active stimulation with a targeted cortical E-field of 8 V m^−1^ to sham stimulation (0 V m^−1^). In the healthy adult population, we systematically varied E-field amplitude and carrier frequency. In the stroke group, participants completed up to 12 sessions of kTMP as part of a pilot intervention study, allowing us to assess if tolerability changes across sessions. Additional datasets come from studies in which kTMP was applied to the dorsolateral prefrontal cortex (DLPFC), superior temporal gyrus (STG), or the cerebellum. Together, these experiments provide a basis for evaluating kTMP tolerability at higher cortical E-field amplitudes across multiple brain regions in healthy adults and patients with chronic stroke.

## 2. Methods

### 2.1. Participants

This research was approved by the University of California, Berkeley Institutional Review Board (Protocol Number 2018–05–11093). The study was conducted in accordance with the principles embodied in the Declaration of Helsinki and in accordance with local statutory requirements. All participants provided written informed consent prior to participation. A total of 143 participants were enrolled in at least one of the 9 experiments (table 1). The cohort was composed of 125 healthy adults (80 women) and 18 patients with chronic stroke (8 women), leading to a total of 433 sessions. No identifiable human subject data are published in this manuscript.

**Table 1.** Experiments, participant populations and stimulation parameters.

Exp	Participant Population	N	kTMP Target	Exp Type	kTMP duration (min)	Total sessions	Exp Cond	Targeted Cortical E-field (V m)	Carrier freq (kHz)	AM freq (Hz)	SAR (W kg^−1^)
1	Healthy adults	43	M1	Sham-comparison	20	86	Active	8	3.5	3, 17–30	0.00017
							Sham	0	N/A	N/A	0
2	Chronic stroke	15	M1	Sham- comparison	5	15	Active	8	3.5	3	0.00017
							Sham	0	N/A	N/A	0
3	Healthy adults	16	DLPFC	Sham- comparison	10	48	Active	7	3.5	N/A	0.00025
							Sham	0	N/A	N/A	0
4	Healthy adults	20	STG	Sham- comparison	∼17	20	Active	6	3.5	3.1	0.00016
							Sham	0	N/A	N/A	0
5	Healthy adults	14	CB	Sham- comparison	10	28	Active	6	3.5	140	0.00029
							Sham	0	N/A	N/A	0
6	Healthy adults	24	M1	Active comparison	10	48	Active	2	3.5	N/A	0.00002
							Active	7	3.5	N/A	0.00026
7	Healthy adults	17	M1	Active comparison	10	68	Active	2	1	N/A	0.00002
							Active	4	2	N/A	0.00008
							Active	7	3.5	N/A	0.00026
							Sham	0	N/A	N/A	0
8	Chronic stroke	7	M1	Feasibility	20	84	Active	8	3.5	3	0.00017
9	Chronic stroke	3	CB	Feasibility	20	36	Active	6	3.5	3	0.00029

Abbreviations: M1 = Primary motor cortex; DLPFC = Dorsolateral prefrontal cortex, STG = Superior temporal gyrus; CB = Cerebellum.

### 2.2. kTMP system

The upgraded kTMP system comprises a high-current amplifier that can deliver currents up to 400 A. The amplifier drives a MagVenture Cool-B70 TMS coil connected to a liquid cooling system. The control system provides the kTMP amplifier with the analog signal to specify the desired kTMP waveform, amplitude, and duration, using hardware-level deterministic timing. Hardware control is accomplished with a National Instruments FPGA card and a PC running the LabVIEW FPGA software. Key safety features of the system include: (1) Monitoring and logging the state of the system through coil temperature and system configuration sensors. (2) Implementing shutdown if the coil temperature ceiling is violated or if the system is out of configuration. (3) Graphical display of the coil temperature. (4) Automatic activation of the cooling system whenever the kTMP system is powered up. (5) Diagnostic coil health check by monitoring coil impedance changes. (6) Monitoring refrigerant temperature and coolant flow.

### 2.3. Experimental design and procedures

The nine experiments reported in this paper were conducted as separate studies to assess different aspects of kTMP (e.g. physiological modulation, behavioral outcomes, patient feasibility). For the purposes of the present analysis, we have organized the data by study type (i.e. sham-comparison, active-comparison, and feasibility) rather than by the chronological order in which they were conducted. Tolerability ratings were collected as a standard outcome measure after every stimulation session across all experiments, allowing us to pool these data to provide a systematic assessment of kTMP tolerability across a wide range of stimulation parameters, brain targets, and participant populations.

The primary objective of each experiment was as follows. Experiment 1 examined the effects of kTMP over M1 on sensorimotor learning in healthy adults [19]. Experiment 2 assessed the tolerability of kTMP in chronic stroke patients in a single session over ipsilesional M1. Experiment 3 assessed the tolerability of kTMP over DLPFC in healthy adults. Experiment 4 investigated the effects of kTMP over STG on auditory perception in healthy adults. Experiment 5 assessed the tolerability of kTMP over the cerebellum in healthy adults. Experiments 6 and 7 assessed the effects of varying kTMP parameters—E-field amplitude and carrier frequency, respectively—on cortical excitability as indexed by motor evoked potentials in healthy adults. Experiment 8 was a multi-session feasibility study examining the effects of 12 sessions of kTMP over M1 on motor impairment and recovery in chronic stroke patients. Experiment 9 was a multi-session feasibility study examining the effects of 12 sessions of kTMP over the cerebellum on motor impairment and recovery in chronic stroke patients.

Experiments 1–5 employed sham-controlled designs, enabling direct comparisons of tolerability between active and sham stimulation. Experiments 2 and 4 used a within-session design in which the participant experienced both active and sham blocks during a single session. Experiments 1, 3 and 5 used a between-session design, where active and sham kTMP were administered on separate experimental days. Experiments 6–7 examined tolerability across active kTMP parameters, while Experiments 8–9 evaluated tolerability across repeated sessions of active kTMP in chronic stroke patients. Participant samples were largely independent across experiments; however, nine healthy adult participants contributed data to more than one study. In these cases, participation in separate experiments was always spaced by at least one month and tolerability ratings were collected independently following each session.

#### 2.3.1. kTMP target locations and E-field modeling

For Experiments 1–2 and 6–8, the M1 stimulation target was determined using a standard ‘hotspot’ procedure employed in many TMS studies [20, 21]. To this end, single-pulse TMS was used to identify the optimal site for activating the contralateral first dorsal interosseous (FDI) muscle. The FDI was chosen because it can be reliably isolated across individuals and exhibits stable threshold values across sessions [22–24]. FDI of the dominant hand was targeted in Experiment 1, FDI of the right hand was targeted in Experiments 6 and 7, and the contralesional FDI was targeted in Experiments 2 and 8. In all experiments, the identified FDI ‘hotspot’ was registered with the Brainsight neuronavigation system (Rogue Research), ensuring consistency of stimulation location across experimental sessions. For E-field simulations we selected C3 as the target location.

To target left DLPFC in Experiment 3, we selected F3 based on the international 10–20 EEG system [25, 26]. To target left STG in Experiment 4, we specified the MNI coordinates (−62 −8 −2) [27], with the location registered to each participant’s head with the Brainsight system. The right cerebellar target in Experiments 5 and 9 was located manually, positioning the coil 2 cm inferior to the inion and 4 cm to the right of the inion. This location was selected to minimize the distance to cerebellar grey matter while also minimizing the amount of occipital stimulation [28].

E-field simulations for magnetic stimulation were performed using SimNIBS version 4.5 [29]. In all simulations, we employed the standard ‘Ernie’ head model, along with the default SimNIBS mesh parameters and isotropic electrical conductivity values for each tissue type. A MagVenture Cool-B70 coil model was placed tangentially over the stimulation targets, with the coil orientation directed toward the contralateral orbit for M1, anteriorly for DLPFC, and inferiorly for the STG and cerebellar targets. Coil center and pose were adjusted to minimize the distance between coil center and target while avoiding collision with the head model. Maximum E-field magnitudes were thresholded at the 99.9th percentile to avoid numerical artifacts at tissue boundaries.

Because simulations were performed using a standard head model rather than individualized anatomy, actual cortical E-field magnitudes will vary across participants. Stimulation intensities were therefore set conservatively, with the simulated E-field in the standard head model calibrated slightly below each nominal target to reduce the likelihood that the intended maximum E-field was exceeded across the range of expected inter-individual anatomical variation.

#### 2.3.2. Subjective rating of stimulation

Immediately following sham or active stimulation, participants were asked three questions to assess expected stimulation-related sensations and tolerability, with the framing of the questions based on prior studies with TMS [8]: 1) *‘Did you experience any annoyance from the stimulation?’; 2) ‘Did you experience any muscle twitches from the stimulation?’; 3) ‘Did you experience any pain from the stimulation?’* Each question was rated on an 11-point scale (0 = no sensation; 2 = just noticeable; 4 = noticeable but not aversive; 6 = somewhat uncomfortable; 8 = very uncomfortable; 10 = intolerable). For the question related to muscle twitches, participants were instructed that this could include incidental muscle activation in the scalp, face, or neck, as well as twitches in the contralateral body when stimulating over motor cortex.

#### 2.3.3. Blinding

Due to electromagnetic forces, the coil and other system components produce a sound at twice the carrier frequency, with the loudness of this sound proportional to the square of the output current [30]. To mask this sound, the PC controlling the kTMP system generated an auditory tone during sham conditions that matched the frequency and intensity of the tone produced during active kTMP stimulation. The experimenters and participants wore foam earplugs during active or sham kTMP stimulation. To further ensure double-blind conditions, experiments incorporated the following safeguards. Condition order was randomized for each participant by a researcher not involved in data collection. The experimenter entered a randomly assigned code corresponding to the experimental condition; this person was not involved in programming experimental parameters. These safeguards ensured that the experimenter administering the session and collecting tolerability ratings remained blind to condition assignment throughout data collection.

Experimenters and participants remained blinded to condition assignments until all data collection were complete. In experiment 1, participants were asked at the end of the last session whether they thought they had received sham or active stimulation.

### 2.4. Safety monitoring and biophysical exposure

#### 2.4.1. Device-related adverse event monitoring

Device-related adverse reactions were defined in accordance with [13] as any untoward and unintended response that occurs during or immediately following a stimulation session. Participants were not systematically followed beyond the session to assess delayed adverse events; however, none were spontaneously reported.

#### 2.4.2. Seizure monitoring

When the upgraded kTMP system enabled electric fields up to 8 V m^−1^, we conducted an initial safety assessment using continuous electromyography (EMG) monitoring. In a pilot study with healthy adults (*n* = 12, 8 V m^−1^ cortical field, 5 min), EMG was recorded from hand muscles (FDI and abductor digiti minimi) and monitored in real-time by an experienced observer for any activity indicative of excessive cortical excitation, including muscle discharges, rhythmic bursting or sustained activity above resting baseline [31]. No formal quantitative threshold was pre-specified, consistent with the exploratory nature of this initial safety assessment. Simultaneously, participants were continuously observed for overt motor activity, including facial twitches, changes in facial expression or involuntary muscle contractions. An identical protocol was implemented in our initial stroke patient cohort (*n* = 15).

Based on these initial assessments, subsequent experiments employed visual monitoring only. All participants were continuously observed during stimulation for signs of abnormal motor activity that could indicate excessive cortical excitation.

#### 2.4.3. Specific absorption rate (SAR)

SAR of the head was computed from the simulated E-field as the volume-averaged absorbed power per unit mass,

\begin{equation*}{\mathrm{SAR}} = \frac{1}{V}\int\limits_{{\mathrm{head}}} {\frac{{\sigma \left( r \right){{\left| {E\left( r \right)} \right|}^2}}}{{p\left( r \right)}}{\mathrm{d}}r} \end{equation*}

discretized as a volume-weighted sum over the tetrahedral elements of the head mesh. Tissue-specific electrical conductivities *σ(r)* were taken from the default SimNIBS isotropic values, and mass densities *ρ(r)* from the IT’IS Foundation tissue-properties database [32]. A factor of one-half was applied to convert the peak amplitude of the sinusoidal E-field to its time-averaged value; for amplitude-modulated waveforms, the SAR was approximated using a factor of one-fourth. The E-field magnitudes included in this summation were thresholded at the 99.9th percentile, as previously described.

### 2.5. Statistical analyses

#### 2.5.1. Permutation tests

To evaluate whether tolerability ratings differed between active vs. sham (Experiments 1–5) or between two active kTMP conditions (Experiment 6), we employed paired sign-flip permutation tests. We chose this nonparametric approach after inspecting the distribution of the data. There were a large proportion of zero ratings, leading to highly non-normal distributions that violate the assumptions of standard parametric tests. Permutation tests do not rely on distributional assumptions and instead generate the null distribution of the test statistic by repeatedly reassigning signs of the paired differences. For small sample sizes (e.g. $n {\unicode{x2A7D}} 20$, we performed an exact test by enumerating all ${2^n}$ possible sign-flip combinations. For larger samples, where full enumeration is impractical, we approximated the null distribution using Monte Carlo sampling with ${2^{20}}$ (≈1 million) random permutations, ensuring stable estimates.

Experiment 7 involved four conditions to compare stimulation at different carrier frequencies. A Friedman test was conducted as a preliminary assessment of whether any systematic differences existed across the four conditions. We then proceeded with pairwise comparisons between all condition pairs using paired sign-flip permutation tests. This included comparisons between sham and each active frequency, as well as comparisons between active frequencies.

No correction for multiple comparisons was applied, as the goal was to demonstrate equivalence rather than control Type I error rate associated with detecting differences. In contrast to conventional null hypothesis significance testing, equivalence testing requires demonstrating that observed differences fall within a prespecified margin of practical indifference. Applying conservative multiple comparison corrections in this context would impose more stringent statistical criteria, but at a cost of reducing sensitivity to detect equivalence.

#### 2.5.2. Equivalence testing

In addition to traditional hypothesis testing, we conducted equivalence testing to evaluate whether active conditions were perceptually indistinguishable from sham within a predefined margin. Equivalence testing differs from standard null hypothesis significance testing in that it assesses whether observed differences are small enough to be considered practically negligible [33]. For our tolerability ratings, the scale was structured such that each integer value reflected a qualitatively distinct perceptual state: a rating of 0 indicated no sensation, while a rating of 2 indicated a just noticeable sensation. As a conservative assumption, we adopted a criterion whereby a one-point change was taken to represent the threshold for transitioning between qualitatively meaningful perceptual categories. On this basis, we defined an equivalence margin of ±1 rating unit, corresponding to 10% of the total scale range, such that differences falling within this boundary were interpreted as perceptually equivalent. While this margin was derived from the qualitative structure of the scale, it is consistent with prior NIBS studies that have defined equivalence bounds [34, 35]. To assess equivalence, we calculated 95% confidence intervals of the mean paired differences between active and sham conditions using nonparametric bootstrapping (10,000 resamples with replacement). If the entire bootstrapped confidence interval fell within the ±1 margin, the active and sham conditions were considered perceptually equivalent. This bootstrap-based approach provides a distribution-free method of equivalence testing that is robust to the zero-inflated, non-normal nature of the rating data.

## 3. Results

kTMP was well tolerated with no device-related adverse reactions occurring in any of the 433 sessions. EMG monitoring in initial experiments (*n* = 27) revealed no artifacts or discharges during stimulation. No participant in any experiment exhibited overt motor activity, facial twitches, or involuntary muscle contractions during any of the sessions. Whole-head SAR values across all experimental conditions are reported in table 1; the maximum SAR across all conditions was 0.00029 W kg^−1^, four orders of magnitude below the FDA regulatory limit of 3.2 W kg^−1^. Coil temperature remained within safe operating limits throughout all sessions.

### 3.1. Blinding assessment

**Figure 1.**
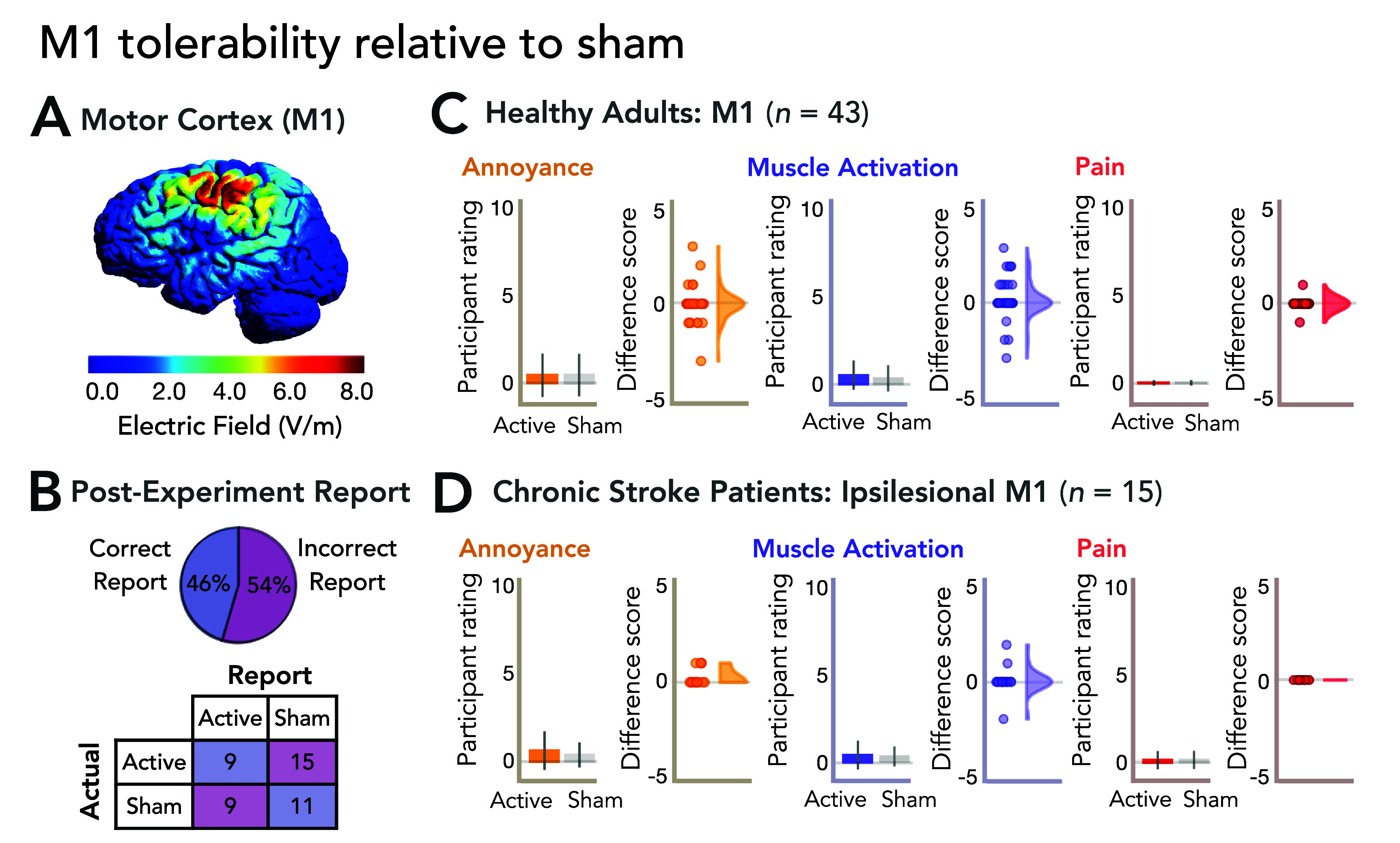
kTMP tolerability over M1 in healthy adults and chronic stroke patients. (A). Simulation of E-field distribution and magnitude for M1 target location using SimNIBS [29]. (B). Blinding assessment in healthy adults (Exp 1) who received 20 min sham and active stimulation over M1 in two sessions with the order counterbalanced. At the end of the second session, participants indicated if they had received active or sham kTMP. Discrimination did not differ from chance (*p* = 0.52). (C). Tolerability ratings for healthy adults (Exp 1) receiving active (8 V m^−1^) versus sham (0 V m^−1^) kTMP. For each measure, left panel shows mean ratings with error bars representing the standard deviation, and right panel shows paired differences (active minus sham) for each participant. (D). Tolerability ratings for chronic stroke patients (Exp 2) receiving ipsilesional M1 stimulation. Format as in panel (C).

In Experiment 1, healthy adults completed two sessions: one with active kTMP stimulation (8 V m^−1^; figure 1(A)) applied over M1 and one with sham stimulation (0 V m^−1^). To assess the effectiveness of blinding, without revealing the existence of a sham condition, participants were asked only at the end of the second session to indicate whether that session involved active or sham stimulation. Of the 44 participants included in the analysis, 20 (45.5%) correctly identified their second-session condition—a performance that did not differ from chance (binomial test, *p* = 0.52, 95% CI [30.4%, 61.2%]; figure 1(B)). When analyzed by condition, participants correctly identified sham in 11/20 cases (55.0%) and active stimulation in 9/24 cases (37.5%), with no significant difference between conditions (*χ*^2^ test, *p* = 0.24). These results demonstrate that participants were unable to reliably distinguish active from sham stimulation, confirming successful blinding.

### 3.2. Tolerability of active vs. sham stimulation

**Figure 2.**
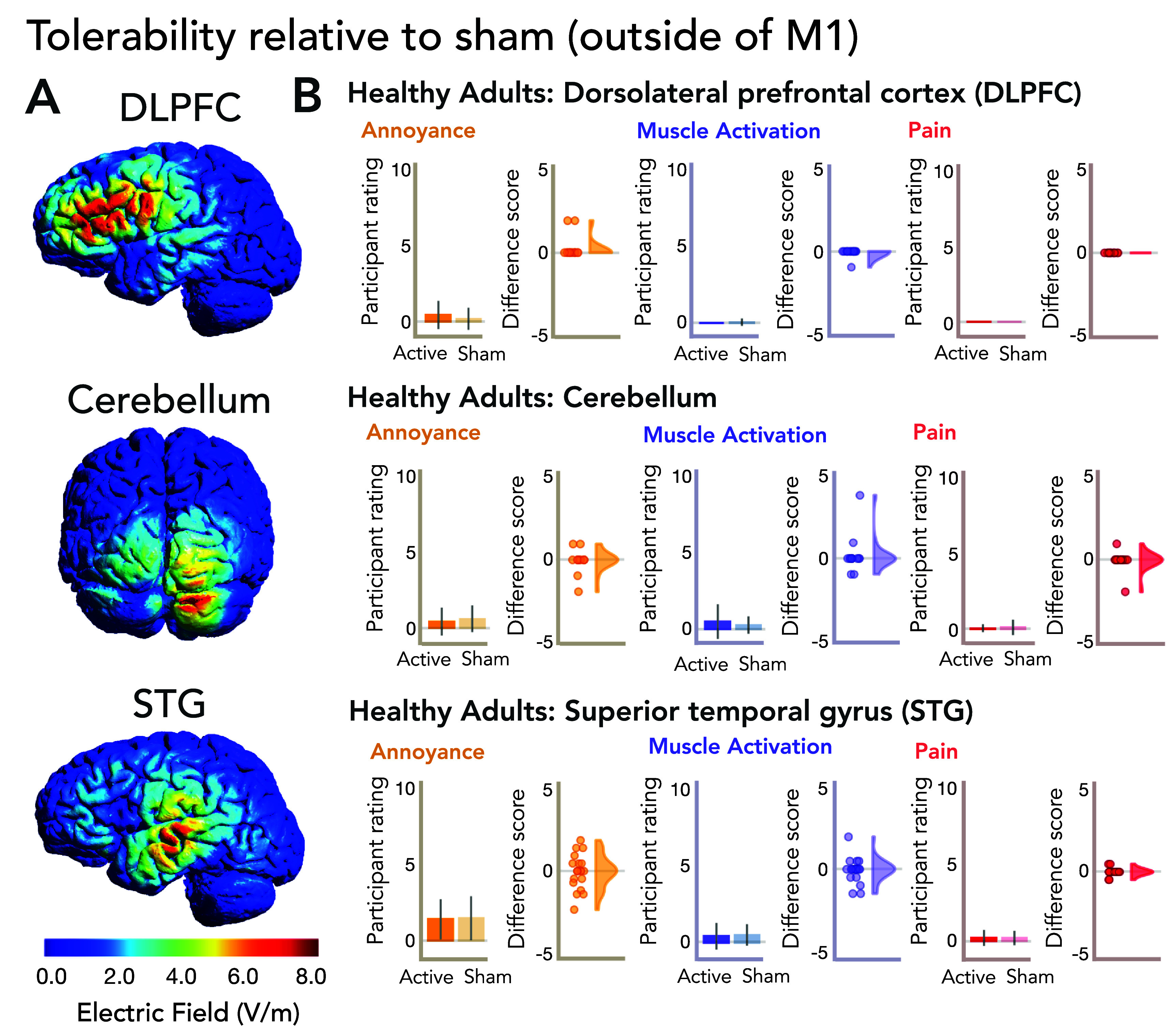
kTMP tolerability outside of M1. **A.** Simulation of E-field distribution and magnitude for target locations using SimNIBS [29]. **B.** Tolerability ratings for healthy adults receiving active and sham kTMP over the DLPFC, cerebellum and STG. For each measure, left panel shows mean ratings with error bars representing the standard deviation, and right panel shows paired differences (active minus sham) for each participant. Across all experiments, annoyance, muscle activation, pain showed no significant differences between active and sham conditions (permutation tests, all *p’s* $ {\unicode{x2A7E}} $ 0.13), and confidence intervals fell within the ±1 equivalence margin.

To probe the subjective experience of kTMP stimulation, participants were asked three questions concerning annoyance, experience of muscle activation, and experience of pain using an 11-point scale (0 = no sensation, 10 = intolerable). Modal ratings for both healthy adults and patients with chronic stroke were zero for stimulation over M1 on all three questions (figures 1(C) and (D)). For both groups, mean ratings for active and sham were below 1 (range: 0.02–0.64) where a rating of 0 was no sensation and a rating of 2 was barely noticeable. A similar pattern was observed for the three other stimulation locations, DLPFC, STG, and cerebellum: Modal ratings in response to both active and sham were zero for all three questions (figure 2) and mean ratings were below 1 (range: 0.00–0.51) for muscle activation and pain, and below 1.5 for annoyance (range: 0.18–1.46).

Mean differences between active and sham stimulation remained low (table 2; range: 0.00–0.27) and permutation tests indicated no evidence of differences between sham and active kTMP over M1 on ratings of perceived annoyance, muscle activation, or pain in healthy adults and patients with chronic stroke. The same observation held in the healthy adults when the coil was positioned to target DLPFC, STG, and cerebellum (table 2; Permutation *p*-value; range 0.13–1.00).

**Table 2.** Permutation tests and equivalence testing of active vs. sham stimulation ratings comparison of active conditions.

Target	Sensation	Mean difference (Active—Sham)	95% Bootstrap CI	Permutation p-value
M1: Healthy adults	Annoyance	−0.02	[−0.27, 0.23]	1.00
	Muscle activation	0.16	[−0.14, 0.45]	0.40
	Pain	0.00	[−0.07, 0.07]	1.00

M1: Chronic stroke patients	Annoyance	0.27	[0.07, 0.53]	0.13
	Muscle activation	0.07	[−0.33, 0.47]	1.00
	Pain	0.00	[0.00, 0.00]	1.00

DLPFC: Healthy adults	Annoyance	0.25	[0.00, 0.62]	0.50
	Muscle activation	−0.06	[−0.19, 0.00]	1.00
	Pain	0.00	[0.00, 0.00]	1.00

Cerebellum: Healthy adults	Annoyance	−0.07	[−0.43, 0.29]	1.00
	Muscle activation	0.21	[−0.29, 0.93]	0.86
	Pain	−0.07	[−0.43, 0.21]	1.00

STG: Healthy adults	Annoyance	−0.05	[−0.54, 0.41]	0.88
	Muscle activation	−0.07	[−0.40, 0.25]	0.77
	Pain	0.00	[−0.10, 0.10]	1.00

To assess perceptual equivalence, we tested whether active stimulation was indistinguishable from sham within an equivalence margin of ±1 rating unit. Our rating scale had anchors at 0 (no sensation) and 2 (just noticeable sensation), with intermediate values taken to represent transitions between these subjective states. We chose the ±1 margin to ensure that equivalence would only be established if differences remained below the threshold for crossing into a distinct perceptual category. On all three subjective ratings, the bootstrapped 95% confidence intervals for active-sham differences consistently fell within this equivalence boundary (see table 2; 95% Bootstrap CI). These results indicate that participants’ subjective experience did not differ in a perceptually meaningful way between active and sham for our three measured dimensions.

We also compared ratings of subjective experience across active stimulation conditions. Varying the E-field magnitude (2 V m^−1^ vs 7 V m^−1^) produced no reliable differences in annoyance, or perceived muscle activation or pain (mean differences $ {\unicode{x2A7D}} $ 0.29; all permutation *p*’s $ {\unicode{x2A7E}} $ 0.50). The bootstrap 95% CI for these comparisons were within the ±1 equivalence margin for muscle activation and pain and only slightly exceeded the margin for annoyance [−0.42, 1.08]. This appears to be driven by a single participant whose annoyance rating was more than two standard deviations above the group mean. When this outlier was excluded, the CI fell below the +1 boundary.

Similarly, no differences emerged when testing different kTMP carrier frequencies. The Friedman test revealed no omnibus effect of frequency on any of the three ratings (all *p*’s $ {\unicode{x2A7E}} $ 0.46). Pairwise permutation tests confirmed that (i) sham did not differ from any active frequency (1000, 2000, or 3500 Hz; mean differences $ {\unicode{x2A7D}} $ 0.24; all permutation *p*’s > 0.50), and (ii) active frequencies did not differ from each other (mean differences $ {\unicode{x2A7D}} $ 0.29; all permutation *p*’s > 0.50). For both sets of comparisons, the bootstrap 95% CIs for mean differences were contained within the ±1 equivalence margin. In summary, ratings on measures of tolerability were comparable across all stimulation frequencies as well as between sham and active conditions.

### 3.3. Tolerability across multiple sessions

We conducted two feasibility studies (Experiments 8 and 9) to assess the effect of kTMP on motor recovery in stroke patients with chronic hemiparesis. As part of the protocol, each participant completed 12 sessions of active kTMP stimulation, with the target of stimulation over M1 in seven patients and the target over cerebellum in three patients. All participants completed the full protocol, with no withdrawals or dropouts. To evaluate tolerability across repeated sessions, we obtained ratings on our three scales after each session. For the M1 group, mean ratings remained low across all 12 sessions: annoyance (*M* = 0.14, range: 0.00–0.41), muscle activation (*M* = 0.03, range: 0.00–0.17), and pain (*M* = 0.05, range: 0.00–0.08) on a 0–10 scale. In the cerebellum group, all three patients reported zero ratings for these measures in all sessions. These findings provide preliminary evidence of good tolerability across repeated sessions, although confirmation in larger samples is needed.

## 4. Discussion

Prior work from our group has demonstrated that kTMP effectively modulates cortical excitability [18]. Here we show that kTMP is highly tolerable across a wide range of stimulation parameters and scalp positions in healthy adults and chronic stroke patients. Across 433 sessions, subjective ratings on measures of annoyance, perceived muscle activation and pain were low with no significant differences between active and sham stimulation. Indeed, equivalence testing confirmed that active and sham conditions were perceptually indistinguishable on these dimensions. Blinding assessment further validated that participants could not reliably distinguish between active and sham stimulation. This perceptual equivalence between active and sham stimulation makes kTMP well-suited for double blind studies in basic research and translational settings.

### 4.1. Safety profile and parameter constraints of kTMP

Beyond subjective tolerability, kTMP also demonstrates a favorable objective safety profile. A key metric for electromagnetic safety is the SAR, which quantifies the rate at which tissue absorbs electromagnetic energy, providing an industry-wide metric to limit heating and prevent thermal injury. Maximum SAR across all kTMP conditions was 0.00029 W kg^−1^, four orders of magnitude below the FDA regulatory limit of 3.2 W kg^−1^. Coil heating also remained well within International Electrotechnical Commission safety standards; with active cooling, surface temperatures stayed below the 43 °C threshold for safe, indefinite skin contact. No seizures occurred and no seizure precursors such as overt motor activity, including facial or limb muscle contractions, were observed in any of the studies. The 8 V m^−1^ targeted cortical E-field is well below the minimum threshold required for direct neuronal activation (∼28 V m^−1^) or seizure induction in nonhuman primates using pulsed stimulation (∼77 V m^−1^) [36, 37]. Although the continuous nature of kTMP could theoretically raise concerns about cumulative effects distinct from pulsed methods such as rTMS, seizure threshold increases markedly above 10–25 Hz, supporting the safety of kilohertz-range stimulation [38]. Therefore, these findings suggest a low risk of seizure induction in healthy adults and individuals with chronic stroke from kTMP stimulation, at least within the range of parameters tested in this study. Taken together, these objective safety characteristics, combined with the perceptual equivalence to a true sham condition (0 V m^−1^), suggest that kTMP may enable access to a broader subthreshold stimulation space while maintaining favorable tolerability and safety margins.

Our current IRB protocol sets an upper bound of 8 V m^−1^ at the cortical surface and restricts stimulation to 20 min per session, either delivered in a single continuous block or divided into shorter epochs. These limits were established conservatively and somewhat pragmatically, guided largely by the hardware capabilities of our upgraded kTMP system and by precedents from other NIBS modalities (e.g. tES, rTMS), rather than by any evidence of approaching safety boundaries. The safety data presented here, including extremely low SAR values, minimal coil heating, and the absence of any subjective discomfort even at the maximum tested parameters, suggest that these protocol limits reside within a robust safety margin. As experience with kTMP accumulates, we anticipate relaxing these constraints to some extent, enabling longer or more intensive stimulation paradigms, including accelerated protocols that require extended total stimulation time.

### 4.2. Limitations

This study has several limitations. Formal blinding assessment was conducted only in Experiment 1. Because sham-controlled experiments involved repeated exposure to both active and sham stimulation—either across multiple sessions or across multiple stimulation blocks within a session—we prioritized subjective tolerability ratings collected immediately after each stimulation condition over post-session blinding checks. This decision was made for two reasons. First, asking participants at the end of each session or stimulation block whether they believed they had received active or sham stimulation risked alerting them to the existence of a sham condition, potentially compromising blinding in subsequent sessions or blocks. Second, blinding assessments administered after repeated exposure require participants to compare stimulation experiences across multiple sessions or blocks, introducing retrospective memory demands that may reduce the reliability of such judgments. Immediate post-stimulation tolerability ratings avoid both of these concerns and provide a direct, condition-specific measure of perceptual equivalence on our targeted dimensions. Nevertheless, the absence of formal blinding assessments in the multi-session and multi-block experiments is a limitation, as tolerability equivalence provides only an indirect proxy for blinding success. Future studies should seek to incorporate formal blinding checks in a manner that does not compromise experimental integrity; for example, by administering a forced-choice question at the conclusion of the final session only, as was done in Experiment 1.

Annoyance ratings showed greater variability than pain or muscle-activation ratings, partly because participants interpreted ‘annoyance’ inconsistently. Some participants based it solely on physical stimulation (which was uniformly low) whereas others also considered the effect of the auditory tone produced by the kTMP system or the masking noise. Moreover, variability in earplug fit likely contributed to individual differences in tone audibility. As a result, annoyance may represent a less specific and less reliable index of stimulation-related tolerability, reflecting a combination of sensory and contextual factors rather than physical effects alone. Importantly, the acoustic properties of the sound (frequency and intensity) were matched between active and sham conditions. As such, any annoyance attributable to sound should affect both conditions equally and would not systematically bias the active–sham comparison. Pain and muscle-activation ratings remained at floor levels across all experiments, indicating consistently high physical tolerability. In addition, while tolerance data were likewise encouraging in the chronic stroke cohort (*n* = 18), including participants who completed multiple sessions (*n* = 10), the overall sample remains modest. Furthermore, because sensory perception may be altered following stroke, the prespecified equivalence margin of ±1 rating unit may not reflect the same perceptual threshold observed in healthy adults. Larger clinical studies and longer treatment protocols will be needed to establish tolerability across broader patient populations and over extended periods of repeated use.

## 5. Conclusions

The demonstrated perceptual equivalence to sham and consistently high tolerability across a wide parameter space distinguish kTMP as a promising modality for both basic neuroscience and translational research. An important next step will be to assess tolerability alongside treatment adherence and completion rates in future studies, as these outcomes can be influenced by discomfort in existing NIBS protocols. If confirmed in future larger clinical trials, these advantages should reduce the risk of participant unblinding, enhancing the reliability of efficacy assessments.

## Data Availability

The data cannot be made publicly available upon publication because they contain sensitive personal information. The data that support the findings of this study are available upon reasonable request from the authors.
